# Characterization and functional analyses of the human HTR1A gene: 5’ regulatory region modulates gene expression in vitro

**DOI:** 10.1186/s12863-018-0708-6

**Published:** 2018-12-29

**Authors:** Xue Wu, Feng-ling Xu, Mei Ding, Jing-jing Zhang, Jun Yao, Bao-jie Wang

**Affiliations:** School of Forensic Medicine, China Medical University, No. 77 Puhe Road, Shenbei New District, Shenyang, 110122 China

**Keywords:** Human HTR1A gene, Promoter, Transcriptional regulation, 5-HT1A receptor, Schizophrenia

## Abstract

**Background:**

The serotonin neurotransmitter (5-HT) and its receptors have important roles in neuropsychiatric disorders such as schizophrenia. The aim of this study was to investigate the functional sequences of the 5′ regulation region of the human HTR1A gene to explore the effects on the expression of the 5-HT1A receptor.

**Methods:**

Fourteen recombinant pGL3-basic vectors containing deletion fragments of the HTR1A gene regulatory region were transfected with HEK-293 and SK-N-SH cells. The relative chemiluminescence intensities of different length fragments were analyzed. The JASPAR software was used for the prediction of transcription factors.

**Results:**

In the HEK-293 cells, the relative chemiluminescence intensity of the − 1649 bp to − 1550 bp (ATG + 1) fragment was significantly different. Two inhibitory activity regions were found in the − 1409 bp to − 1381 bp and − 1196 bp to − 1124 bp fragments, which might be bound to the GATA or SOX10 transcription factors as predicted by the JASPAR software. In addition, the fragments located from − 1124 bp to − 1064 bp and from − 908 bp to − 722 bp up-regulated protein expression. Only the sequence from − 1550 bp to − 1409 bp demonstrated a difference in luciferase expression in the both cell lines. According to the results of the 5’-UTR truncated vectors, there was a repression region at the distal end of the 5’-UTR, an enhancer region might be present at the proximal end of the transcription start site.

**Conclusions:**

Although the functional sequences of the HTR1A gene regulatory region were confirmed, the regulatory factors and functional components require further investigation.

## Background

Many studies have shown that the serotonin system plays an important role in neuropsychiatric disorders such as epilepsy [1], depression [2, 3], and schizophrenia [4, 5]. Abnormalities of the serotonin neurotransmitter (5-HT) have also been observed in these disorders [6]. Interestingly, it is the serotonin receptors that determine the function of 5-HT. Among the subtypes of the serotonin receptors, the 5-HT1A receptor has attracted much attention because it has the widest distribution and highest concentration in the human brain [7, 8]. Previous studies have shown that certain brain regions, such as the prefrontal cortex and the cerebellar vermis, of patients with schizophrenia had an abnormal quantity of 5-HT1A receptors [9–11].

In the neural cells, the basal expression of the 5-HT1A receptor is controlled by a proximal promoter and an upstream inhibitory region in the HTR1A gene 5′ regulatory region [12]. Recent research identified a negative regulatory area containing a novel dual repressor element located in the upstream promoter region (− 1590 bp to − 1517 bp) of the HTR1A gene in the rat. This region, which appears to be essential for the regulation of the 5-HT1A receptor expression, is approximately 80% homologous to the human HTR1A gene [13, 14]. Although some other functional fragments were confirmed in the same study, the localization of the regulatory sequence was inaccurate and imprecise, primarily due to the large range of fragments. Therefore, the functional characterization of the 5′ regulatory region of the HTR1A gene, which encodes the 5-HT1A receptor, is poorly understood.

Additionally, most studies have focused on 2 polymorphisms--rs6295 (−1019G/C) in the promoter region and rs878567 (*287 T/C/G) located in the 3’-UTR of the human HTR1A gene. It has been reported that the G allele of rs6295 up-regulates the expression of the 5-HT1A receptor and reduces 5-HT levels, which may contribute to severe depression [15], schizophrenia [16], and other neuropsychiatric disorders. Our previous research involved in the haplotype study also demonstrated that the G allele of rs6295 increased the gene expression and might contribute to the susceptibility of schizophrenia [17]. Conversely, other researchers have posited that this single nucleotide polymorphism (SNP) exerts its effects by changing the binding capacity of the transcription factors instead of directly regulating gene expression [18]. Another study also suggested that rs6295 did not impact the expression of HTR1A gene [19]. Meanwhile, some reports have proved that rs878567 locus is significantly associated with schizophrenia [16]. Notably, our previous case-control experiment revealed that the human HTR1A gene polymorphism rs113195492 (−1068G/A) G allele might be a susceptibility gene in female paranoid schizophrenia [20]. However, the subsequent haplotype did not confirm that this polymorphism had a significant impact on protein expression [17].

At present, studies on the functional sequences and polymorphisms of the HTR1A gene 5′-regulatory region involved in schizophrenia were not comprehensive and the conclusions remained controversial. Therefore, we constructed recombinant vectors containing different length sequences in the HTR1A promoter and 5’-UTR regions: we used the dual luciferase reporter assay to explore specific functional areas and their effects on gene expression.

## Methods

### Construction of target-fragment pGL3 recombinant vectors

Primers (Table 1) and the introduction of B*gl*II, H*ind*III, or N*he*I restriction endonuclease cleavage sites at the 5′ end of the corresponding primers were used to amplify the target fragments. The longest target fragment was located in the human HTR1A gene from − 1902 bp to + 99 bp (ATG + 1). The fragment was used as an amplification template for other truncated bodies to synthesize 12 recombinant vectors with 5′ deletion. Then, the fragment located from − 1124 bp to + 99 bp (ATG + 1) was used to amplify the 2 target sequences missing the 3′ end (Although the two target fragments were truncated from the 3′ end, the transcription start site remained [21]). The purified target genes were cloned into a pGM-T vector using the pGM-T Ligation® Kit (TIANGEN, Biotech, Beijing, China), and the recombinant vectors were screened by transformation and sequencing. Finally, pGM-T vectors were subcloned into pGL3 vectors (Promega, Madison, Wisconsin, USA; Catalog number: E1751), and the recombined pGL3 vectors were selected in the same manner to complete eukaryotic cells transfection [17].

**Table 1:** Primer sequences of the target fragments containing the cleavage sites

Target fragments	Primer sequences^a^
B*gl*II -HTR1A(− 1902)F	5’-GAAGATCTCAGGTGAAAGTGTTAGCCTAGC-3’
B*gl*II -HTR1A(− 1781)F	5’-GAAGATCTCGAGCGCAAGAGGGCACTA-3’
B*gl*II -HTR1A(−1649)F	5′- GAAGATCTTCGGCATAACGGTTTCCAGATG-3’
B*gl*II -HTR1A(−1550)F	5’-GAAGATCTTCTGAGATTAAGAGAGGCTAGCCG-3’
B*gl*II -HTR1A(−1409)F	5’-GAAGATCTATCCCTGAATCTACTAGCCACA-3’
B*gl*II -HTR1A(−1381)F	5’-GAAGATCTTGGGAAGTGGCAGTGTCACTG-3’
B*gl*II -HTR1A(− 1235)F	5’-GAAGATCTATTTCGTTCTCTCCCGGTTCC-3’
B*gl*II -HTR1A(−1196)F	5’-GAAGATCTTCACAGGCAATATTCTCCCTGAG-3’
N*he*I -HTR1A(−1124)F	5’-CTAGCTAGCTTGTCGTCGTTGTTCGTTTG-3’
B*gl*II -HTR1A(−1064)F	5’-GAAGATCTGTGTAATGGTGCGAGAACGG-3’
B*gl*II -HTR1A(−908)F	5’-GAAGATCTGGATGCTGACACGATTTAAGAAT-3’
B*gl*II -HTR1A(−722)F	5’-GAAGATCTAGACTTGAATGCAAAGACGCTG-3’
H*ind*III -HTR1A(+ 99)R	5’-CCCAAGCTTGTCGGAGATACCAGTAGTGTT-3’
H*ind*III -HTR1A(−119)R	5’-CCCAAGCTTCAGGAAGTTCTTACTGCTTC-3’
H*ind*III-HTR1A(−372)R	5’-CCCAAGCTTAAATATCTAGAACCGAGAAGCC-3’

^a^The 5′ end of the primer sequences contained the B*gl*II, H*ind*III, and N*he*I restriction enzyme cleavage sites

The numbers in brackets represent the 5′ end position in the HTR1A gene as ATG + 1. F, Forward; R, Reverse

### Cell culture

A total of 14 recombinant vectors were transfected into human embryonic kidney cell HEK-293 and human neuroblastoma cell SK-N-SH lines (Human embryonic kidney cell HEK-293 and human neuroblastoma cell SK-N-SH were purchased from the Chinese Academy of Sciences cell bank and catalog numbers were GNHu43 and TCHu51, respectively.). HEK-293 cells were incubated in HyClone® DMEM high-glucose medium containing 10% fetal bovine serum (Thermo Fisher Scientific, Waltham, Massachusetts, USA); they were maintained in 5% CO_2_ + 95% mixed air at 37 °C. SK-N-SH cells were treated with KeyGEN BioTECH® DMEM high-glucose medium with 0.110 g/L sodium pyruvate containing 15% fetal bovine serum under the same conditions. Transfection experiments were performed when the cell density reached 90% or more [17].

### Transient transfection and dual luciferase reporter analysis

Cells with densities of 90% were inoculated into 24-well plates at a concentration of 2 × 10^5^ cells per well. After incubation for 36 to 48 h, 14 recombinant vectors were co-transfected with the internal control Renilla luciferase-expressing vector (The internal control vector pRL-TK (Promega) minimizes the experimental variability caused by the differences in cell type and quantity, transfection efficiency or cell lysis efficiency.) using the Lipofectamine® 2000 reagent (Invitrogen, Carlsbad, California, USA). The cells were incubated for 24 h, and then the cell lysates were collected and the expressions of the Firefly luciferase (LUC) and the Renilla luciferase (TK) were detected. Each sample had three replicate wells in each experiment. Need to repeat three complete experiments [17].

### Statistical analysis

The LUC/TK value represents the relative fluorescence intensity. Nine LUC/TK values were obtained for each recombinant vector. The relative chemiluminescence intensity of each recombinant vector is expressed as a mean ± standard deviation. The one-way analysis of variance (ANOVA) and the least significant difference (LSD)-T test were performed using SPSS version 20.0 software. One-way ANOVA was used to compare the differences among all vectors. The LSD-T test was only used to compare adjacent fragments, which could reduce the occurrence of the I type of error. A *p*-value less than 0.05 represented a statistically significant difference in relative chemiluminescence intensities [22].

### Transcription factor prediction

Bioinformatics analysis was performed on functional fragments that had a significant effect on protein expression. JASPAR software was used to predict the transcription factors (http://jaspar.genereg.net/cgi-bin/jaspar_db.pl) [23]. A transcription factor and DNA sequence matching degree of 85% was considered perfect.

## Results

### Construction of pGL3 recombinant vectors with different length target fragments in the 5′ regulatory region of the HTR1A gene

**Fig. 1:**
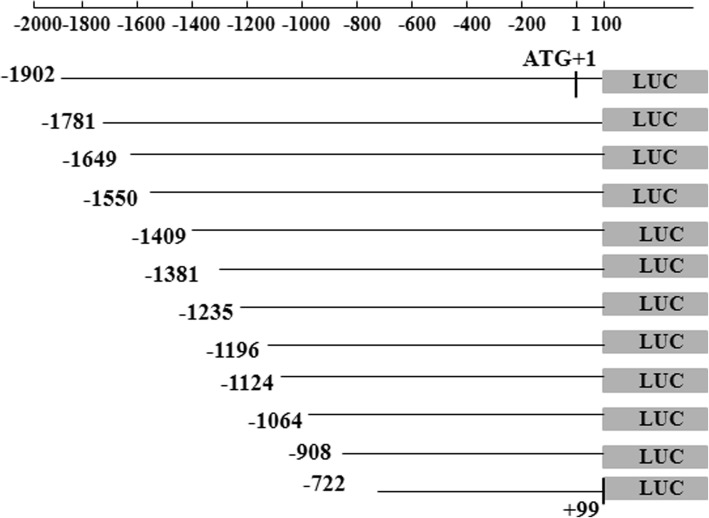
The 12 constructed pGL3 recombinant vectors contained the desired fragment. The locations of the target fragments of the 12 recombinant vectors, which contained a deletion from the 5′ end of the HTR1A gene regulatory region, are shown. The longest target fragment was located in the − 1902 bp to + 99 bp region as ATG + 1. The 3′ end positions of the other amplified fragments were unchanged. All fragments removed from the 5′ end were approximately 100 bp to 200 bp in length

**Fig. 2:**
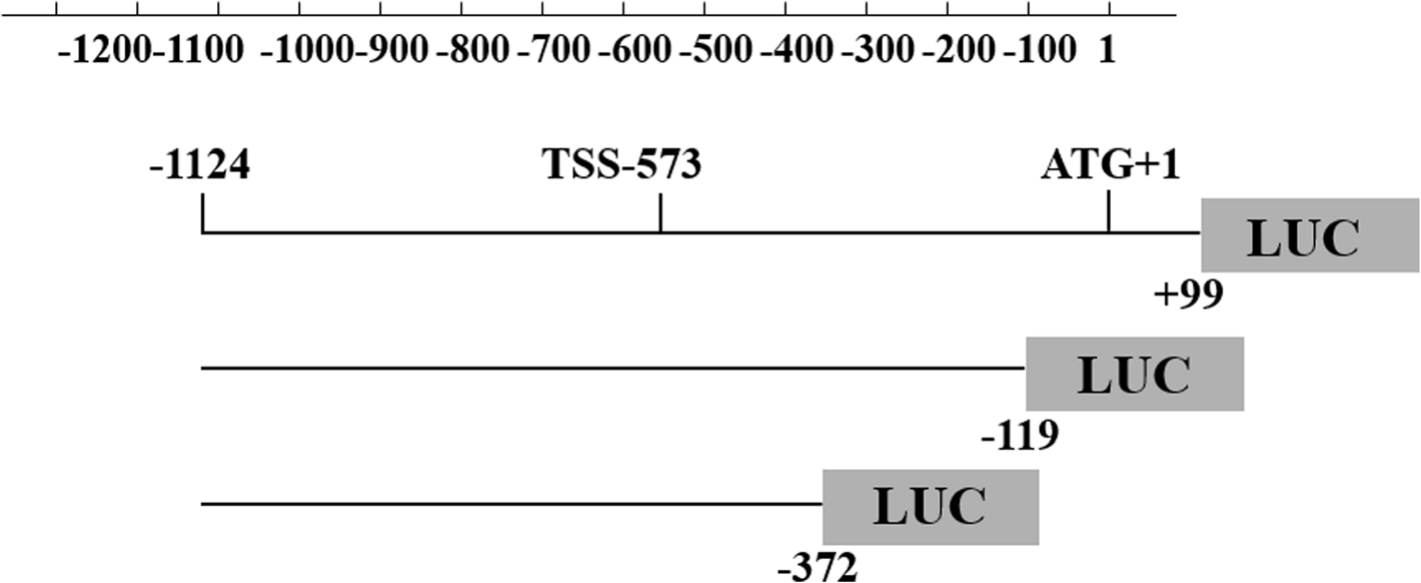
The pGL3 recombinant vectors contained the desired fragment located in the HTR1A gene 5’-UTR region. The detailed position of the fragment in the HTR1A gene 5’-UTR region is shown. The − 1124 bp to − 119 bp and − 1124 bp to − 372 bp sequences were generated from the constructed − 1124 bp to + 99 bp sequence

A total of 14 different length sequences of the HTR1A gene were cloned into pGL3-Basic vectors. Details of the inserted fragments are presented in Figs. 1 and 2.

### Comparison and analysis of relative fluorescence intensities of 14 recombined vectors of the HTR1A gene regulatory region

**Fig. 3:**
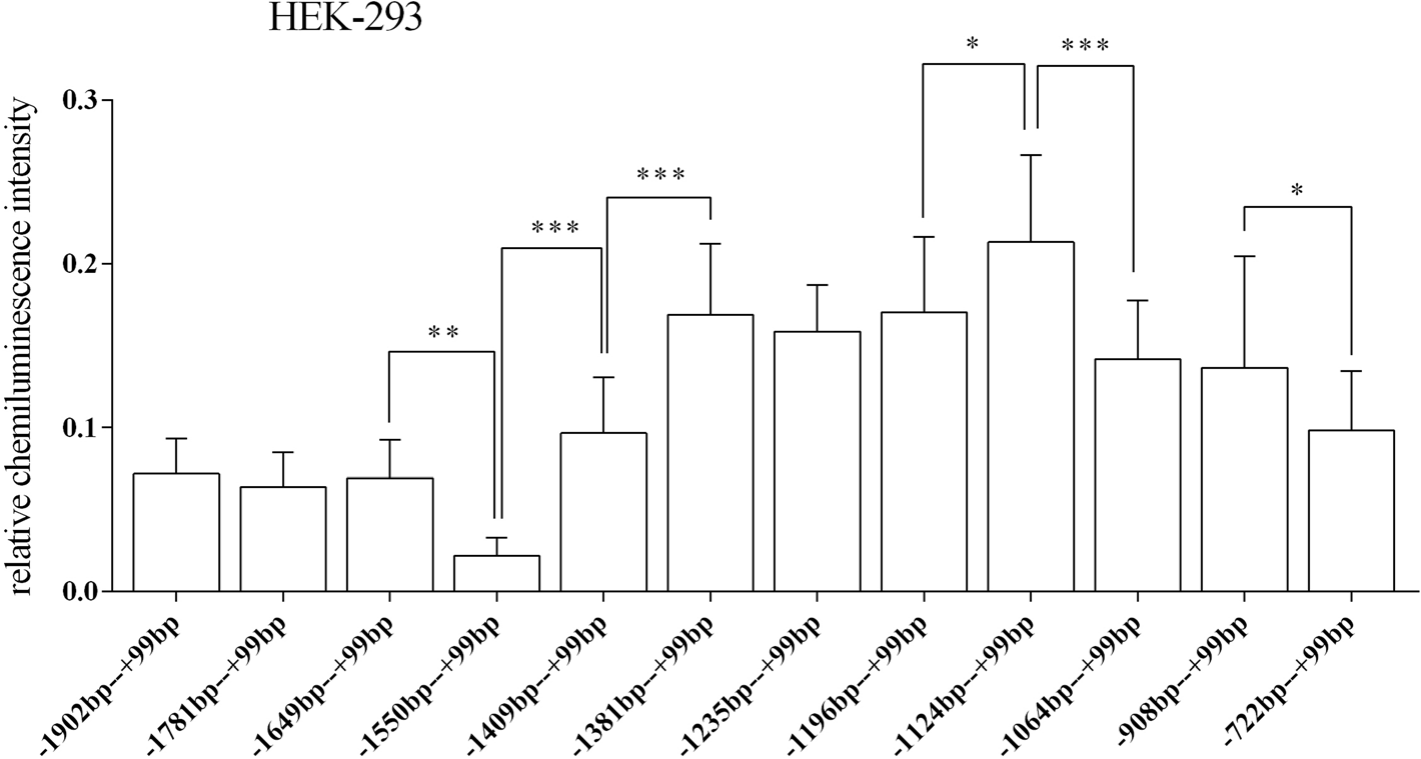
Relative fluorescence intensities of the 12 recombinant vectors with the 5′ end deletion in the HEK-293 cell line. There were significant differences in the relative fluorescence intensities between several fragments: − 1649 bp to + 99 bp, − 1550 bp to + 99 bp, − 1409 bp to + 99 bp and − 1381 bp to + 99 bp. There were also 2 regions with significant regulatory effects on gene expression: − 1196 bp to − 1124 bp and − 1124 bp to − 1064 bp. The relative fluorescence intensity is expressed as the mean ± standard deviation. The difference in relative fluorescence intensity between adjacent sequences was determined by the least significant difference-T test. * 0.02 < *p* < 0.05, ** 0.001 < *p* < = 0.02, *** *p* < = 0.001

**Fig. 4:**
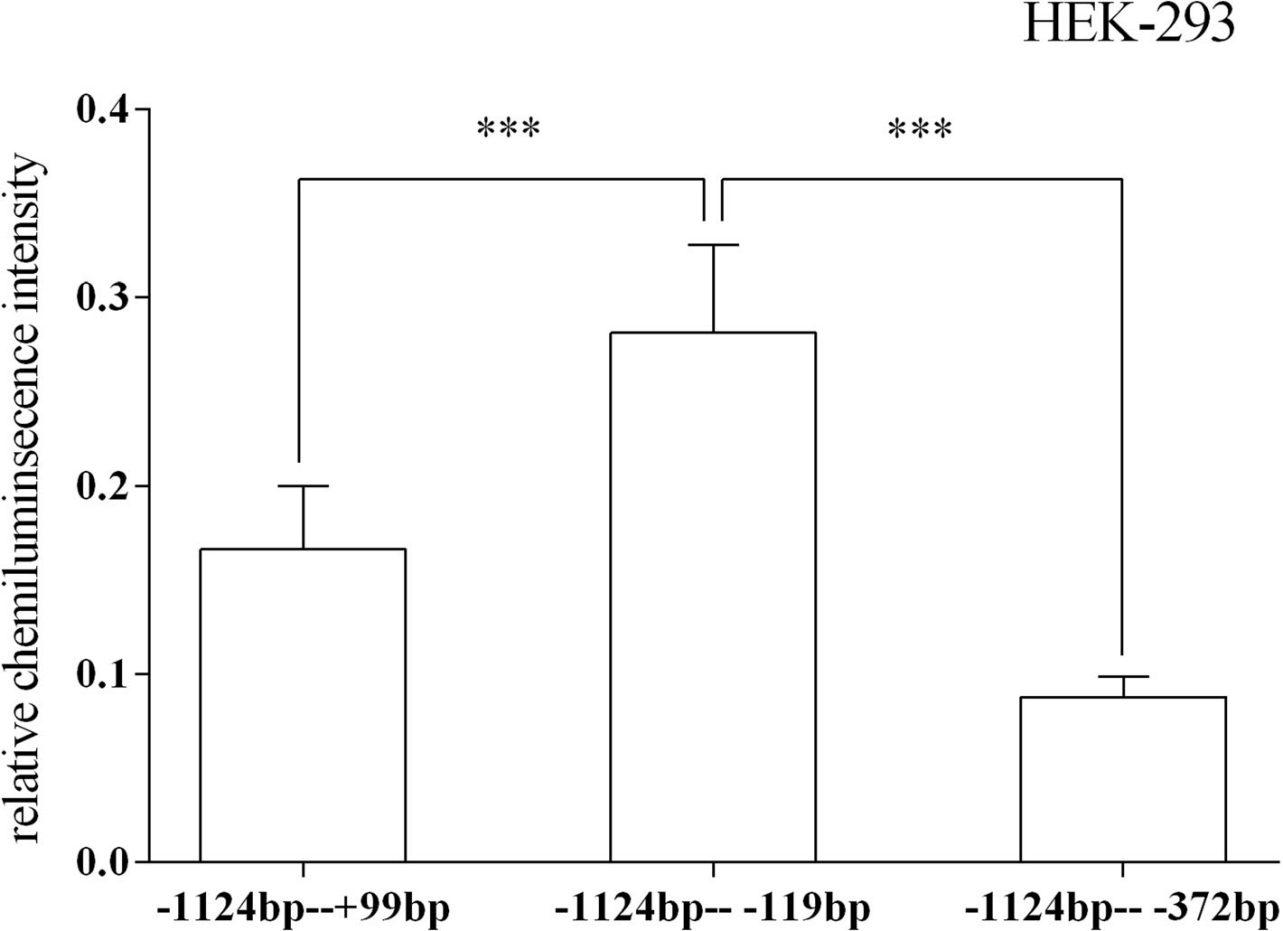
Relative fluorescence intensities of the recombinant vectors in the 5’-UTR of the HTR1A gene in the HEK-293 cells. The sequences from − 119 bp to + 99 bp and from − 372 bp to − 119 bp significantly regulated the luciferase expression. According to the change in relative fluorescence intensity, a transcriptional suppressor was identified between − 119 bp and + 99 bp, and one enhancer might be located between − 372 bp and − 119 bp. *** *p* < = 0.001

A total of 14 recombinant plasmids were transfected into 2 cell lines to verify the roles of different sequences in the HTR1A gene regulatory region. In the HEK-293 cells, the fragment − 1124 bp to + 99 bp (ATG + 1) had the highest relative chemiluminescence intensity. The relative chemiluminescence intensities of all recombinant vectors containing the target fragments were statistically significant with the *p* = 0.000 (degree of freedom (11, 96); *F* = 19.294). A comparison of the relative chemiluminescence intensities of 3 pairs of 4 fragments (− 1649 bp to + 99 bp, − 1550 bp to + 99 bp, − 1409 bp to + 99 bp and − 1381 bp to + 99 bp) were significantly different: − 1649 bp to + 99 bp vs. -1550 bp to + 99 bp (*p* = 0.01); − 1550 bp to + 99 bp vs. -1409 bp to + 99 bp (*p* = 0.000); − 1409 bp to + 99 bp vs. -1381 bp to + 99 bp (*p* = 0.000). Comparisons of the relative chemiluminescence intensities among 3 other fragments (− 1196 bp to + 99 bp, − 1124 bp to + 99 bp, and − 1064 bp to + 99 bp) also revealed significant differences: − 1196 bp to + 99 bp vs. -1124 bp to + 99 bp (*p* = 0.02) and − 1124 bp to + 99 bp vs. -1064 bp to + 99 bp (*p* = 0.000). Additionally, there was a significant difference between the relative chemiluminescence intensities of the − 908 bp to + 99 bp and − 722 bp to + 99 bp fragments (*p* = 0.036) (Fig. 3). Finally, the relative chemiluminescence intensities of the 3′ deletion sequences (− 1124 bp to + 99 bp, − 1124 bp to − 119 bp and − 1124 bp to − 372 bp) were also significantly different: − 1124 bp to + 99 bp vs. -1124 bp to − 119 bp (*p* = 0.000) and − 1124 bp to − 119 bp vs. -1124 bp to − 372 bp (*p* = 0.000) (Fig. 4).

**Fig. 5:**
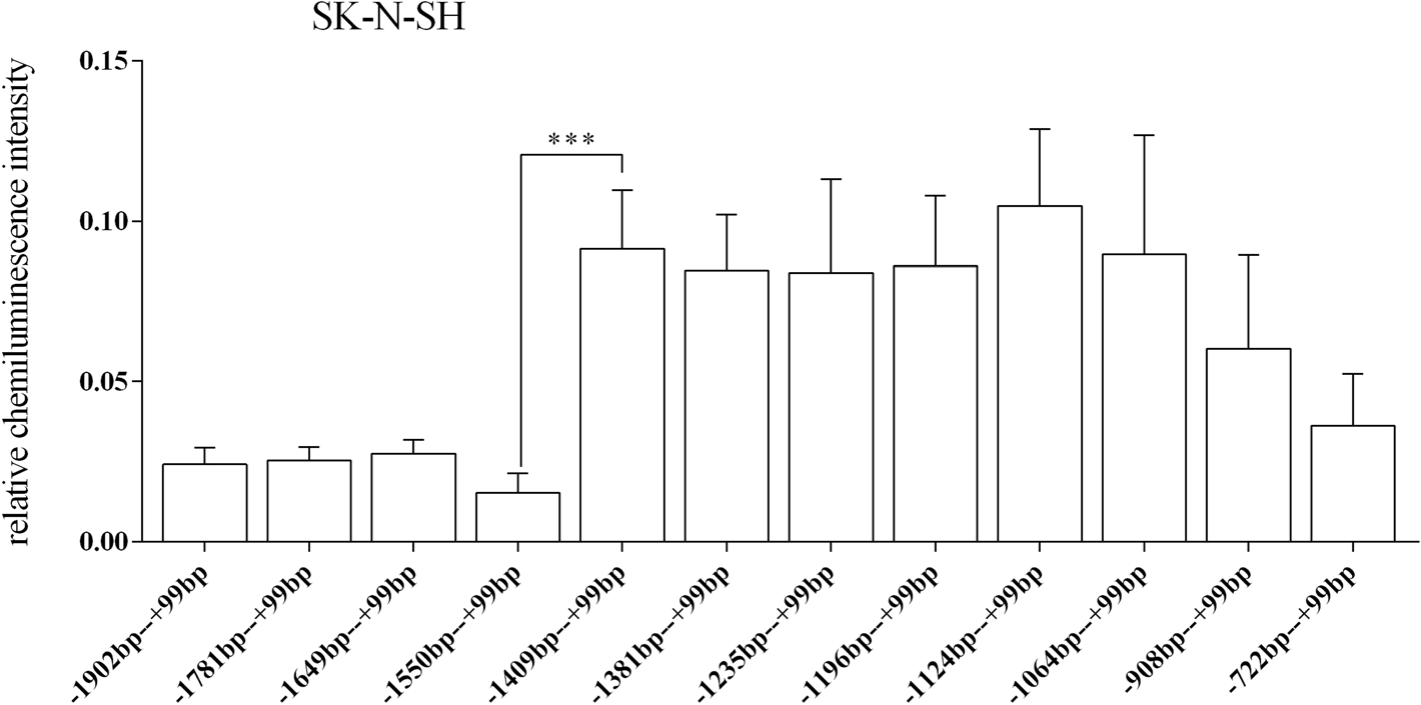
Relative fluorescence intensities of the 12 recombinant vectors with the 5′ end deletion in the SK-N-SH cell. Only one fragment (− 1550 bp to − 1409 bp) demonstrated a significant difference in relative fluorescence intensity. However, there might be several regulatory region located between − 1409 bp and − 1124 bp and between − 1124 bp and − 908 bp. Still, the relative fluorescence intensities were only slightly different. *** *p* < = 0.001

**Fig. 6:**
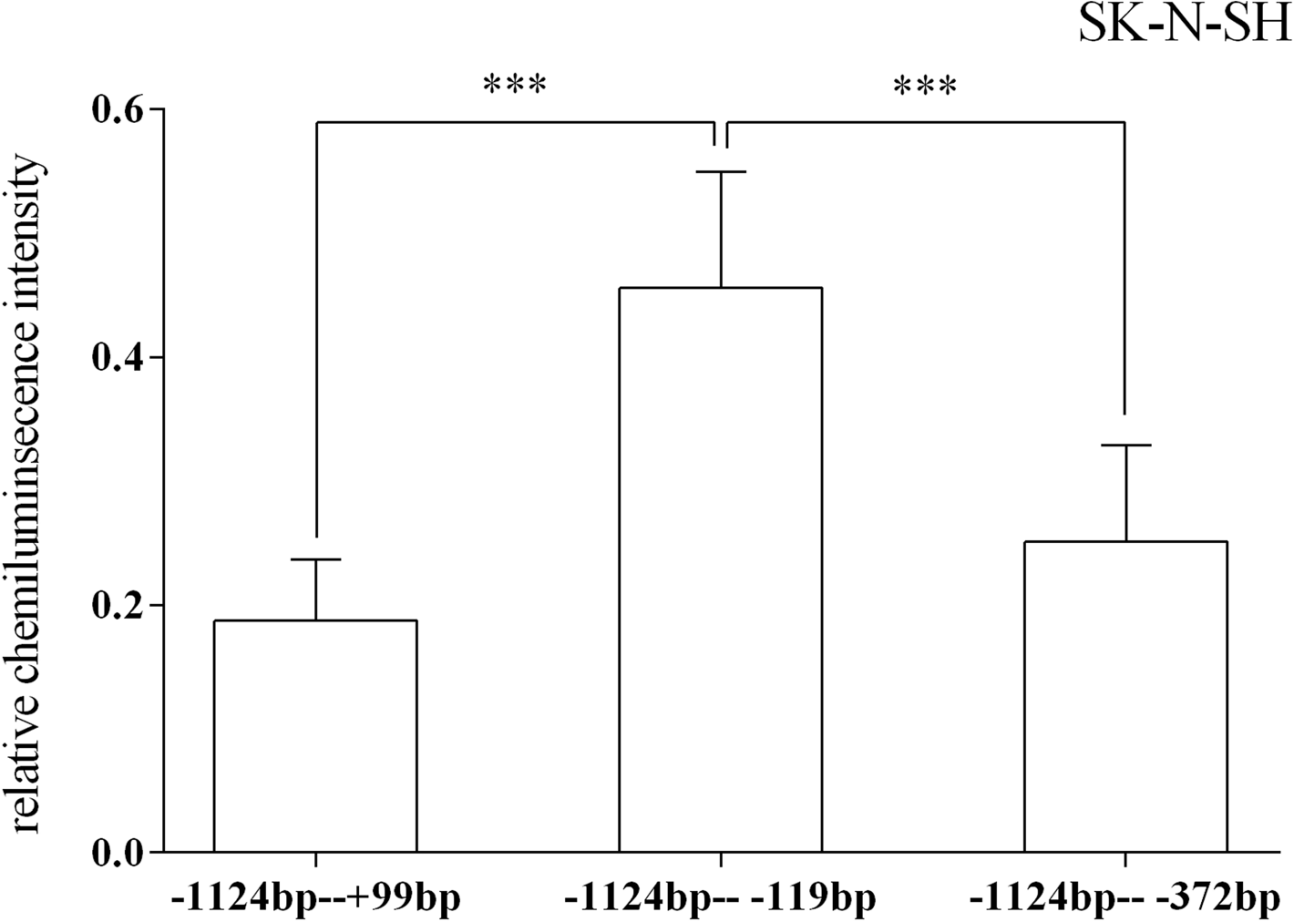
Relative fluorescence intensities of the recombinant vectors in the 5’-UTR of the HTR1A gene in the SK-N-SH cells. In the 5’-UTR region of the HTR1A gene, the relative fluorescence intensities of the 3 recombinant vectors were significantly different, which was similar to the trends observed with the SK-N-SH and HEK-293 cells. The relative fluorescence intensity of the − 1124 bp to − 372 bp fragment was higher than that of the − 1124 bp to + 99 bp fragment in the SK-N-SH cells but lower in the HEK-293 cells. *** *p* < = 0.001

In the SK-N-SH cell line, the results of one-way analysis of variance showed that the regulatory activities of all target fragments showed significant differences and the *p* value was 0.000 (degree of freedom (11, 96); *F* = 7.370). However, the only statistically significant difference was observed between the 5′ deletion fragments − 1550 bp to + 99 bp and − 1409 bp to + 99 bp (p = 0.000) (Fig. 5). Moreover, for the target fragment of 3′ deletion, the relative chemiluminescence intensities of − 1124 bp to + 99 bp, − 1124 bp to − 119 bp and − 1124 bp to − 372 bp fragments were significantly different: − 1124 bp to + 99 bp vs. -1124 bp to − 119 bp (p = 0.000) and − 1124 bp to − 119 bp vs. -1124 bp to − 372 bp (p = 0.000). Additionally, the trend in luciferase expression of the 14 truncated vectors was similar to that of the HEK-293 cells, but the relative chemiluminescence intensities were generally lower in the SK-N-SH cells (Fig. 6).

### Prediction and screening of transcription factors

**Fig. 7:**
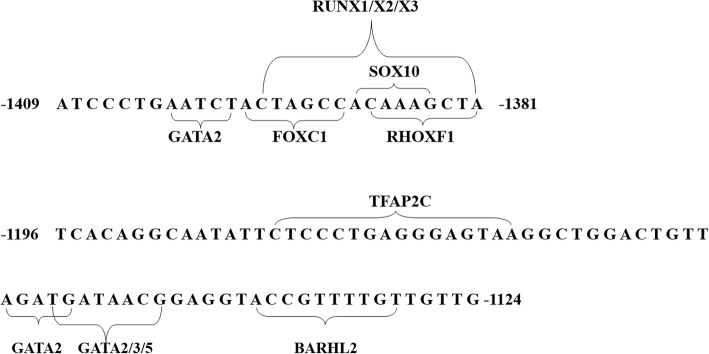
Transcription factor prediction of functional fragments. The predictive results of transcription factor binding to the − 1409 bp to − 1381 bp fragment or the − 1196 bp to − 1124 bp fragment are shown. The numbers in front of the fragments represent the 5′ end position in the HTR1A gene. The bases in parentheses represent those recognized by the transcription factors with a perfect match of 85%

**Fig. 8:**
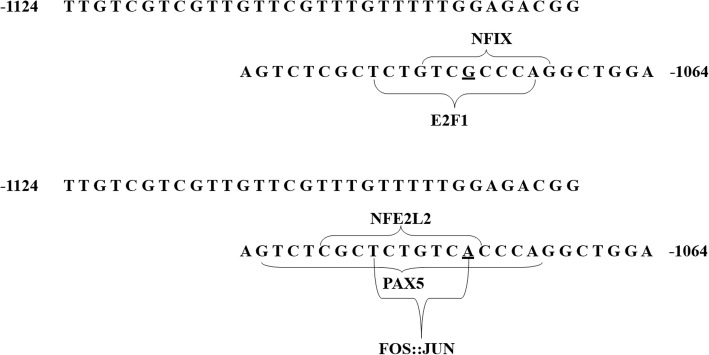
Transcription factor prediction of rs113195492. This figure shows the rs113195492 G and A alleles bound to transcription factors. When the G allele was converted to an A, the potential binding transcription factors changed from E2F1 and NFIX to PAX5, NFE2L2, and FOS::JUN. Underlined portions represent the 2 alleles

The results of JASPAR software showed that the functional sequences − 1409 bp to − 1381 bp and − 1196 bp to − 1124 bp might be recognized by the transcription factors SOX10, GATA or RUNx family and TFAP2C, which might play a crucial role in the development of the nervous system (Fig. 7). For the sequence − 1124 bp to − 1064 bp including the polymorphism rs113195492, we predicted that when the G allele was converted to an A, the potential binding transcription factors changed from E2F1 and NFIX to PAX5, NFE2L2, and FOS:: JUN (Fig. 8).

## Discussion

In order to study the functional sequence of the regulatory region of the HTR1A gene, 14 recombinant pGL3 vectors with different length segments were constructed, transfected into 2 cell lines and analyzed by the luciferase reporter assay. The results showed that, in the HEK-293 and SK-N-SH cell lines, the relative chemiluminescence intensity was significantly increased when the − 1550 bp to − 1409 bp fragment was removed. The luciferase expression of the truncated sequence located from − 1649 bp to − 1550 bp was decreased in both cell lines, but a larger decrease was noted in the HEK-293 cells. These results suggest that there might be an enhancing regulatory factor in the − 1649 bp to − 1550 bp region and that an inhibitory region might be located in the − 1550 bp to − 1409 bp region. As far as we know, there was an inhibitory region of 107 bases or 71 bases (− 1624 bp to − 1517 bp and − 1590 bp to − 1519 bp [ATG + 1]) of the human or rat HTR1A gene: this region including two copies of the dual repressor element (DRE) and a repressor element-1 (RE-1**)** binds 2 transcription factors (Freud-1 and REST) and plays a potentially important role in the silencing of the HTR1A gene [13, 14]. Therefore, we essentially verified that the sequence located at − 1624 bp to − 1517 bp did posses a suppressor region. Importantly, it appeared that this regulatory area could be reduced to a narrower range (the overlap of the sequence − 1624 bp to − 1517 bp and − 1550 bp to − 1409 bp). Hence, the 33 bases between − 1550 bp and − 1517 bp seem to be the core of the inhibitory region.

By comparing the relative chemiluminescence intensities in the HEK-293 cells, a functional region was identified that had inhibitory activity in the − 1409 bp to − 1124 bp sequence. We then truncated this region further and demonstrated that there were 2 functional inhibitory regions located between − 1409 bp and − 1381 bp and between − 1196 bp and − 1124 bp, which suggests that there might be some inhibitory transcription factors binding at these bases. The JASPAR analysis led to the theory that there may be some transcription factors, such as GATA, SOX10 and RUNX1/X2/X3, binding the sequence from − 1409 bp to − 1381 bp. Meanwhile, the transcription factors most likely to recognize the sequence − 1196 bp to − 1124 bp might be the TFAP2C and GATA families (Fig. 7). A search of the PUBMED database revealed that GATA and SOX10 have been used as common transcription factors to regulate the expression of multiple genes and TFAP2C might play an important role in the development of the nervous system. Related studies have demonstrated that the overexpression of the transcription factor GATA in the hippocampus leaded to a decrease in spinal synaptic density and synaptic-related gene expression, which ultimately resulting in the depressive behaviors [24]. Additionally, GATA also played an important role in the Parkinson’s disease related genes [25]. For the SOX10 and RUNx-related transcription factors, they had a regulatory effect on the peripheral and central nervous system [26, 27]. We hypothesized that the predicted transcription factors might be the essential reasons of regulatory functions of the − 1409 bp to − 1381 bp and − 1196 bp to − 1124 bp sequences. However, subsequent experiments are needed to further prove.

According to our previous haplotype study, the C allele of the rs6295 locus significantly down-regulated protein expression and the G allele of rs113195492 slightly up-regulated gene expression [17]. In this study, we further explored the effects of these 2 SNPs on gene expression by constructing pGL3 recombinant vectors that lacked the rs113195492 G allele or the rs6295 C allele at the 5′ end. In the HEK-293 cells, the relative chemiluminescence intensity of the − 1124 bp to + 99 bp fragment was significantly different than the − 1064 bp to + 99 bp fragment. The deleted region, containing the rs113195492 (−1068G/A) locus, significantly down-regulated protein expression. These results are consistent with our previous study. However, compared to the − 1064 bp to + 99 bp fragment, the deleted region of the rs6295 C allele at the − 908 bp to + 99 bp fragment did not show an effect on protein expression [12]. Some studies have shown that the effect of rs6295 on gene expression results mainly from the combination of transcription factors such as Deaf-1 and Hes5 [28], so we speculated that rs113195492 may also be combine with several transcription factors. Therefore, the possible bindings of the A and G alleles of rs113195492 were predicted separately, one by one. The results showed that when the G allele was converted to an A, the potential binding transcription factors changed from E2F1 and NFIX to PAX5, NFE2L2, and FOS::JUN (Fig. 8). Unfortunately, neither the role of the 2 polymorphic sites nor the role of specific fragment lengths could be proved precisely. Thus, more research is needed to clarify the exact mechanisms of these 2 SNPs on protein expression.

Considering that the 5’-UTR region plays important roles in the stability of mRNA, ribosome transport, and initiation regulation of translation [29], 2 recombinant vectors containing 3′ end deletion fragments (− 1124 bp to − 119 bp and − 1124 bp to − 372 bp) were constructed. Comparison with the − 1124 bp to + 99 bp fragment, which showed the strongest transcriptional activity, revealed that the relative chemiluminescence intensities of the 3 truncated vectors were significantly different between the 2 cell lines. Related studies reported that the − 79 bp, − 115 bp and − 350 bp sites of the HTR1A gene (ATG + 1) bind 2 regulatory factors---Pet-1 and NFκB, while both enhance regulatory effects on the HTR1A gene [6]. However, in this study, the truncated fragment including the 2 elements located in the − 119 bp to + 99 bp fragment showed an inhibitory effect on luciferase expression in the 2 cell lines. We speculated that, on one hand, this conflicting result might be due to differences between species and cell lines, but, on the other hand, some regulatory factors could also have regional and temporal specificity. Related study indicated that Pet-1 transcription factor was highly expressed in the adrenal gland and brain. However, the expression of Pet-1 was lower in the intestines and eyes. Particularly, Pet-1 activated the gene transcription exhibited the beta43’ enhancer and the cell-type dependence [30]. In addition, the expression of Pet-1 in the brain was restricted to the developing or adult 5-HT neurons [31, 32]. Therefore, its effects in other tissues or cells might be quite distinct. By comparing the relative chemiluminescence intensities of the − 1124 bp to − 119 bp fragment and the − 1124 bp to − 372 bp fragment, we confirmed the conclusions of other studies that reported that NFκB enhances gene expression [33, 34]. However, when comparing the effects of − 1124 bp to + 99 bp and − 1124 bp to − 372 bp fragments on luciferase expression, we found that the relative chemiluminescence intensity values were opposite in the 2 cell lines. We believe that the ambiguity of this result might be due to the fact that the regulatory factors in several cell lines might be slightly different.

In this study, we synthesized pGL3 recombinant vectors containing different sequences of the human HTR1A gene regulatory region and more accurately mapped the functional fragments. However, only the base positions of the enhancer regions and the inhibitory regions were determined. The mechanisms of the regulatory factors and functional elements in each region are still unclear. In some regions, transcription factors were also predicted, and subsequent experiments were conducted to demonstrate the binding of these transcription factors and their effects on protein expression. The experimental results were controversial, possibly due to the fact that the experiments were conducted in a single cell lines, which is a limitation to this study.

## Conclusions

By constructing pGL3 recombinant vectors with different length sequences of the HTR1A gene regulatory region, the enhancer regulatory region and the inhibitory regions located between − 1649 bp and − 1550 bp and between − 1550 bp and − 1409 bp, respectively, were identified. Down-regulated elements were also present in the fragments from − 1409 bp to − 1381 bp and − 1196 bp to − 1124 bp. The effects of rs113195492 and rs6295 polymorphisms on protein expression were further confirmed by truncation experiments. The influence of the 5’-UTR region of the HTR1A gene on gene expression was also explored. However, follow-up experiments are needed to identify the transcription factors and other regulatory elements that bind to the HTR1A gene sequences [35].

## Abbreviations

ANOVA: One-way analysis of variance
LSD: Least significant difference
SNP: Single nucleotide polymorphism
